# Integrated insulin-iron nanoparticles: a multi-modal approach for receptor-specific bioimaging, reactive oxygen species scavenging, and wound healing

**DOI:** 10.1186/s11671-024-04024-6

**Published:** 2024-05-30

**Authors:** Komal Attri, Bhupendra Chudasama, Roop L. Mahajan, Diptiman Choudhury

**Affiliations:** 1 Department of Chemistry and Biochemistry, Thapar Institute of Engineering and Technology, Patiala, Punjab 147004 India; 2 Centre of Excellence for Emerging Materials, Thapar Institute of Engineering and Technology, Patiala, Punjab 147004 India; 3 Department of Physics and Material Sciences, Thapar Institute of Engineering and Technology, Patiala, Punjab 147004 India; 4 Department of Mechanical Engineering, Department of Materials Science and Engineering Virginia Tech, Blacksburg, VA 24061 USA

**Keywords:** Protein protected nanoparticles, Insulin, Receptor-targeted bioimaging, ROS, Wound healing, Iron-oxide nanoparticles

## Abstract

Metallic nanoparticles have emerged as a promising option for various biological applications, owing to their distinct characteristics such as small size, optical properties, and ability to exhibit luminescence. In this study, we have successfully employed a one-pot method to synthesize multifunctional insulin-protected iron [Fe(II)] nanoparticles denoted as [IFe(II)NPs]. The formation of IFe(II)NPs is confirmed by the presence of FTIR bonds at 447.47 and 798.28 cm^−1^, corresponding to Fe–O and Fe–N bonds, respectively. Detailed analysis of the HR-TEM-EDS-SAED data reveals that the particles are spherical in shape, partially amorphous in nature, and have a diameter of 28.6 ± 5.2 nm. Additionally, Metal Ion Binding (MIB) and Protein Data Bank (PDB) analyses affirm the binding of iron ions to the insulin hexamer. Our findings underscore the potential of IFe(II)NPs as a promising new platform for a variety of biomedical applications due to their high signal-to-noise ratio, and minimal background fluorescence. The particles are highly luminescent, biocompatible, and have a significant quantum yield (0.632). Exemplar applications covered in this paper include insulin receptor recognition and protection against reactive oxygen species (ROS), harmful molecules known to inflict damage on cells and DNA. The IFe(II)NPs effectively mitigate ROS-induced inflammation, which is a hinderance to wound recovery, thereby facilitating enhanced wound recovery.

**Graphical abstract:**

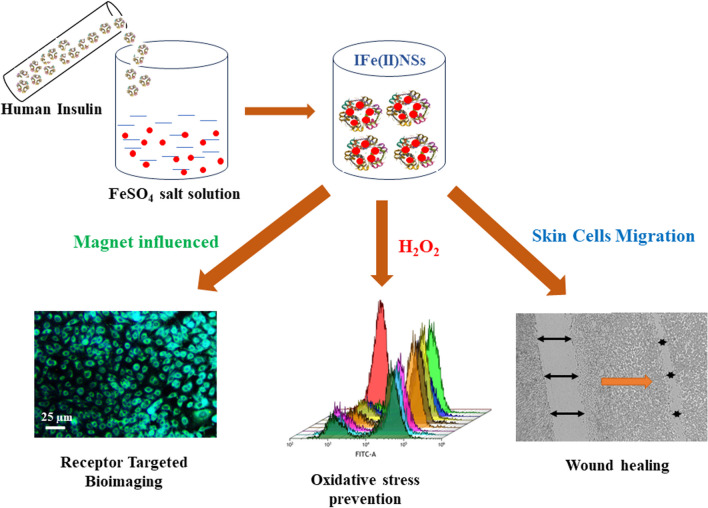

**Supplementary Information:**

The online version contains supplementary material available at 10.1186/s11671-024-04024-6.

## Introduction

Nanotechnology has emerged as a pivotal convergence point for various disciplines, including biology, chemistry, biotechnology, chemical engineering, and pharmaceutical sciences, driving extensive research in a multitude of areas [1, 2]. A key focus has been on the development of novel materials with specific chemical and physical properties of interest. Current investigations are dedicated to creating nano-formulations with diverse shapes and sizes, recognizing that the electronic, optical, and chemical behaviors of these formulations markedly differ from those of bulk materials [3]. Currently, various nanoformulations, including nanoparticles, quantum dots, nanoclusters, quantum clusters, hydrogels, nanofibers, and scaffolds, are being developed for their role in wound healing, targeted drug delivery, and bioimaging applications. This surge in interest is driven by the expanding demands for efficient and cost-effective wound healing [4].

The human skin, the body’s largest organ, serves as a barrier against the entry of microbes. Comprising the epidermis, dermis, and hypodermis layers, along with nerve endings, hair follicles, and hormonal glands, the skin exhibits a complex morphology. Although the skin possesses the remarkable ability to heal itself, this restorative capacity can be impaired during severe damage caused by chemical or physical means [5].

The wound healing process itself is complex and involves four major sequential and overlapping phases: hemostasis, the inflammatory phase, the proliferative phase, and the remodeling phase [6]. Hemostasis initiates clot formation triggered by the activation of platelets, which prevent the entry of microbes and stimulate matrix formation. The inflammatory phase is a complex response initiated by damage and pathogen-associated molecular patterns, along with the release of components from damaged tissues and bacteria. This phase is accompanied by the release of pro-inflammatory cytokines that are crucial for effective healing. The proliferative phase involves the gathering of different healing-promoting factors, such as growth factors, connective tissues, and angiogenesis factors, at the site of injury. The final phase, remodeling, involves the resynthesis of the extracellular matrix, the maintenance of cell numbers through the replacement of dead cells with fast-growing cells, and the formation of collagen, which provides tensile strength [7, 8].

Wound healing becomes challenging and gets exasperated under certain conditions, such as microbial growth, diabetes, and ROS accumulation. Reactive oxygen species (ROS) are produced as incidental byproducts of cellular metabolism, originating from both the electron transport chain (ETC) in mitochondria and the cytochrome P450. The accumulation of Reactive Oxygen Species (ROS) poses a significant challenge as it hinders the effective functioning of macrophages and endogenous stem cells. Additionally, ROS induces endothelial dysfunction and hampers angiogenesis, thereby impeding the regeneration of wound tissue. In addition to ROS produced by the wound itself, bacterial infection further contributes to ROS production, ultimately leading to chronic wound infection by causing damage to endothelial cells and blood vessels [9, 10].

Insulin plays a critical role in wound healing by promoting cell proliferation, collagen synthesis, angiogenesis, and immune system deregulation. Insulin facilitates the healing of wounds by deactivating NFkβP50/P65 and stimulating the synthesis of proteins and lipids. Additionally, it modulates the dynamic balance between pro-inflammatory and anti-inflammatory cytokines [11].

Driven by these advantages, the utilization of insulin protein is on the rise, driven by the abundant presence of insulin receptors on the membranes of all mammalian cells. Notably, there exists a substantial variation in the receptor count, ranging from a mere 40 in erythrocytes to 200–300 × 10^3^ in adipocytes and hepatocytes. It’s worth highlighting that the number of receptors is markedly higher in cancerous cells compared to their normal [12, 13]. Its pivotal function as a growth factor includes aiding chemotaxis and the processes of pinocytosis or phagocytosis by macrophages. Additionally, it promotes the secretion of inflammation-critical cytokines and contributes to re-epithelialization, a crucial element in the wound healing process. Its mechanism involves the transformation of proinflammatory cytokines into anti-inflammatory ones, fostering wound repair and regeneration [4, 14]. The insulin receptor is related to receptor tyrosine kinase transmembrane signaling proteins present on the surface of cells which are responsible for activating the Akt and Erk signaling pathways after binding and improving the wound healing abilities. It also binds to IGF receptors and exhibits anti-inflammatory activity through different signaling pathways, such as Akt and PI3K, which activate cytokine STAT-3 and promote cell growth and angiogenesis. Furthermore, insulin inactivates the TNFα-mediated inflammatory pathway, which is widely known for its role in inactivating the pro-inflammatory cytokines [11, 16].

Sharda et al. synthesized insulin-loaded chitosan nanoformulations for their potential role in burn wound healing by modulating the Nrf-2 pathway [15]. Kaur et al. formulated zinc insulin quantum clusters which were used for wound recovery as well as a monitoring tool [12]. Kaur et al. made insulin-loaded silver nanoparticles and studied them for their wound-healing capacity in the case of normal wounds as well as diabetic wounds both in vivo and in vitro [4]. Nanoformulations play a significant role in wound healing through their unique properties that can enhance the effectiveness of wound treatment. They offer advantages, such as targeted drug delivery, enhanced penetration, protection, and stability of growth factors or sensitive drugs, reduction in both dosage required and toxicity, acceleration of healing processes by promoting cell proliferation and angiogenesis and tissue regeneration, and prevention of infection [16–19].

Among various nano-formulations, protein-based nanoformulations are attracting interest owing to their notable features, including significant biodegradability, easy metabolization, and the potential for surface modification to enhance drug attachment efficiency. These proteins, sourced from plants or animals, such as bovine serum albumin [20], insulin [5], transferrin [21], lactoferrin, etc., are utilized in the synthesis of nano-formulations. The choice of nanoparticles for wound treatment is guided by three essential criteria: their antimicrobial properties, function as effective delivery agents, and contribution to the overall repair process [22, 23].

Nano-formulations incorporating insulin as a protein template show considerable promise in diverse applications such as bioimaging, super-resolution microscopy, and conventional wound healing [24, 25]. The incorporation of insulin protein in nanoformulations serves as a protective agent, offering exceptional stability, biocompatibility, water solubility, and strong fluorescence, making it an ideal nanomaterial for biological applications [26, 27].

Previously, various metal ions, including silver, copper, zinc, nickel, and cobalt, among others, have been employed to create distinct formulations with insulin. Essential metallic elements like calcium, copper, iron, and zinc play indispensable roles in diverse regulatory pathways within the human body, influencing the organized process of wound repair either directly or indirectly [28]. Iron, serving as a component of hemoglobin, plays a pivotal role in hemostasis. In instances of body damage, the release of ferrous ions from hemoglobin contributes to the promotion of blood clotting. Further, it is responsible for promoting collagen synthesis which eventually provides mechanical support to the healing tissue and promotes cell migration across it. It also increased the secretion of vascular endothelial growth factor (VEGF) and Hypoxia-inducible factor (HIF-α). [29]. During hemostasis, zinc, calcium, and iron play crucial regulatory roles in the coagulation cascade, preventing excessive blood loss. In the inflammatory response phase, certain metallic elements like zinc, manganese, and iron are involved in controlling inflammation-related cells, ensuring that the inflammatory response initiates and concludes within an appropriate timeframe [30, 31]. Additionally, iron plays a crucial role in wound healing by modulating fibroblast behavior and facilitating extracellular matrix deposition [29]. Iron also plays a pivotal role in inducing the expression of fibroblast matrix metalloproteinases-1 (MMP-1) [32]. Furthermore, the luminescent properties of iron clusters contribute to their significance as a promising material [33, 34]. Various multifunctional iron-based nanoparticles have been synthesized for bioimaging and therapeutic applications [35]. For instance, iron oxide nanocolloids are increasingly employed to visualize cellular contributions in neuroinflammation using MRI [36]. Similarly, superparamagnetic iron oxide nanoparticles, which exhibit magneto-fluorescence, serve as multicolor imaging probes [37]. Based on the above considerations, we selected to focus our investigation on the synthesis of insulin-based iron nanoparticles due to their remarkable characteristics. We posited that the insulin-based iron nanoparticles hold the potential to serve as effective agents for bioimaging, and wound healing across diverse cell types, while also exhibiting ROS scavenging potential, which is crucial for efficient wound healing as excessive ROS generation impairs healing ability. To test our premise, we undertook a comprehensive characterization of the synthesized nanoparticles using various techniques that provide valuable insights into their physicochemical properties, electrical properties, and morphological features, all of which are essential for their in vitro and in vivo applications. Consequently, we delved into the exploration of the potential of these nanoparticles for fluorescence bioimaging, wound healing applications, and also ROS scavenging activity.

## Materials and methods

### Materials

All chemicals used in the experiment were of analytical / cell culture grade. Ferrous sulfate (FeSO4), sodium hydroxide (NaOH), hydrochloric acid (HCl), formaldehyde, Dulbecco’s Modified Eagle Medium (DMEM) media, Foetal Bovine Serum (FBS), and penicillin–streptomycin were purchased from HiMedia, India. Recombinant human insulin was purchased from Elli Lilly, India. The remaining chemicals were also of analytical grade and purchased from HiMedia, India.

The Human Primary Epithelial Keratinocytes (HEKa cells) ATCC-PCS-200-011 were procured from Himedia, India, and were cultured, maintained, and treated in DMEM containing 5% FBS at 37 °C and 5% CO_2_.

### Preparation of Insulin-iron nanoparticles

Insulin-iron nanoparticles were synthesized using a one-pot synthesis method which is previously documented [38–40]. Initially, a 3.47 M concentration of fresh insulin was taken, then adjusted to the final concentration of 1.82 µM by taking 536 µl of insulin from stock and making the final volume 1.25 ml by diluting in DI water. Then the pH (10.5) was adjusted by using a 1N NaOH solution. This was labeled as solution A. In parallel, salt Solution (B), using FeSO_4_ was prepared by dissolving in DI water in a covered glass vial with a molarity matching the final insulin concentration of 1.82 µM. Then, these two solutions were thoroughly mixed, and the pH was adjusted to a physiological pH of 7.4 using 0.1 N HCl. The resultant solutions were labeled IFe(II)NPs and kept in an incubator for 48 h at 37 °C with agitation at 240 rpm. The final solution was stored at 4 °C for further characterization.

### Morphological and elemental analysis

Dynamic Light scattering (DLS) was employed to estimate the hydrodynamic size of IFe(II)NPs. The sample preparation involved centrifugation at 240 rpm for about 10–15 min, followed by rigorous washing to dispose of any possible impurities. This rigorous processing ensured the generation of high-quality samples needed for DLS, HRTEM, and SAED analyses. To study morphology and atomic packing, we performed High-Resolution Transmission Electron Microscopy (HRTEM) using Talos F200S G2 from Thermo Scientific, along with Selected Area Electron Diffraction (SAED). To investigate the presence of different elements in the sample and their respective percentage, Electron Dynamic Scattering (EDS) was employed using SEM JEOL JSM-6300.

### XPS analysis

To determine the compositional and chemical state of the prepared IFe(II)NPs sample, X-ray Photoelectron Spectroscopy (XPS) was performed using PHI 5000 VersaProbe III. It’s a highly surface-sensitive technique [41].

### Drug loading and release kinetics

To investigate the release kinetics of insulin from the synthesized IFe(II)NPs, a 1 ml sample was subjected to centrifugation at 10,000 rpm for 15 min. The concentration of insulin protein was measured in both the supernatant (unbound protein) and the pellet (bound protein) using the Bradford reagent. Subsequently, the release rate of bound insulin from the pellet was monitored at various time intervals (0, 0.5, 1, 2, 4, 8, 16, 24, 32, 40 h) over 40 h. Absorbance values were recorded at 595 nm and plotted to analyze the release trend of the drug. Bovine serum albumin (BSA) standard curves were employed to interpret the results [42, 43].

### In silico studies

Our focus in silico studies was on predicting binding site residues for different transition metal ions. To this end, we used the Metal Ion Binding (MIB) online server, employing the fragment transformation method. This method involved aligning the protein* S* with a metal-binding template *T*. The structural units, each constituted by N-Cα-C atoms of a given residue, were thoroughly analyzed [44]. The focus of our analysis was on understanding the interactions between the insulin protein and metal ions. It specifically helped us comprehend & visualize the spatial positioning of the metal-binding residues in a 3D space [45]. Also, it allowed us to infer the distance between amino acid residues of the specific protein chains (Chain A & Chain B) and the respective metal ions.

### Study of interactions between insulin and salt solutions using FTIR and Raman spectroscopy

We harnessed the capabilities of the Agilent Cary 600 series FTIR Spectrophotometer to obtain the IR spectrum to infer the functional groups present in IFe(II)NPs. The sample pellet was mixed with potassium bromide (KBr) and scanned from 400 to 4000 cm^−1^ after washing and drying at 37 °C [46]. Surface Enhanced Raman Scattering (SERS) Spectra was taken to determine the structural changes in the protein insulin. The sample preparation, undertaken ten minutes before the measurement, utilized silicon wafers. The samples were subjected to scanning from 500 to 1800 cm^−1^ using the instrument LabRam Hr Evolution Horiba. This instrument is equipped with a detector and microscope, which was used to record the Raman spectra for insulin, FeSO_4,_ and IFe(II)NPs [47].

### Cytotoxicity testing

The viability of HEKa cell line (Human Epidermal Keratinocytes) was assessed through the MTT (3-(4,5-dimethylthiazol-2-yl)-2,5-diphenyltetrazolium bromide) assay. To this end, HEKa cells, having a density of 1 × 10^4^ (per well density), were seeded in 96 well plates and allowed to become confluent up to 70–75%. After this, cells were incubated using three different concentrations (1.5, 7.5, 30, and 60 µM) of IFe(II)NPs, insulin, FeSO_4_, and a mixture of insulin and FeSO_4_. Following a 24 h incubation at 37 °C, MTT (2 mg/ml in 5% ethanol) was added and left for three hours. After this incubation, both the MIT and media were removed from each well. Subsequently, 200 µl dimethyl sulfoxide (DMSO) was added to dissolve the formazan crystals, and absorbance was measured at 570 nm. The inhibition percentage was calculated using the following equation.

1 $$ \% {\text{ inhibition }} = \, \left[ {{1} - \left( {{\text{A}}_{{\text{t}}} /{\text{A}}_{{\text{c}}} } \right)} \right] \, \times { 1}00 $$

where A_t_ is the test substance absorbance and A_C_ control solvent absorbance [47, 48]. While performing the MTT assay, five mutually independent replicate sets were taken for each concentration. The average of all those readings was calculated and plotted along with the error bars based on standard deviation.

### Spectroscopic characterization of IFe(II)NPs

UV–visible spectroscopy was conducted using a Shimadzu UV-2600 spectrophotometer equipped with a 4000 µl quartz cuvette featuring a 1 cm path length, enabling a precise measurement of optical density. It was scanned from 200 to 800 nm. Along with IFe(II)NPs, optical density was also calculated for iron salt solution FeSO_4_ and insulin to discern the difference between Insulin and Insulin-linked metal nanoparticles.

For assessing the fluorescence properties of the sample, fluorescence spectroscopy was performed using Agilent Technologies Cary Eclipse. The excitation wavelength was set at 272 nm, which was coupled with an emission scan spanning from 200 to 800 nm. Both excitation and emission slits were set at 20 mm to measure the fluorescence intensity. Subsequently, the quantum yield was calculated for both insulin and IFe(II)NPs with respect to tyrosine, using the following formula:

2 $$ {\text{Q}}.{\text{Y}}.\left( {\text{S}} \right) \, = \frac{{{\text{Q}}.{\text{Y}}.\left( {{\text{Tyr}}} \right) \, \times {\text{ I }}\left( {\text{S}} \right) \, \times { 1} - {1}0^{{ - {\text{Al}}}} \left( {{\text{Tyr}}} \right) \, \times {\text{ n}}^{{2}} \left( {\text{S}} \right)}}{{{\text{I }}\left( {{\text{Tyr}}} \right) \, \times { 1} - {1}0^{{ - {\text{Al}}}} \left( {\text{S}} \right) \, \times {\text{n}}^{{2}} \left( {{\text{Tyr}}} \right)}} $$

Here, Q.Y.is quantum yield; I is Integrated Emission Intensity; n is the Refractive Index of Solvent; A is the Absorbance at Excitation wavelength; l is the length of absorption cell; Tyr indicates tyrosine as the reference, and S refers to the sample under examination.

### Fluorescence bioimaging

Fluorescence imaging was conducted using the Dewinter fluorescence microscope in this study. Human epidermal keratinocytes adult (HEKa) cells were initially seeded in a 35 mm plate and allowed to reach confluence at 75–80%. Subsequently, these cells were placed on a coverslip and incubated for 24 h. Thereafter, the cells were treated with IFe(II)NPs of 30 µM concentration for 6 h. Followed by washing with PBS, fresh media was added to the plates. Images were taken at a particular wavelength both in the absence and presence of a magnet to assess fluorescence [49].

### Effect of IFe(II)NPs on recovery of normal wound healing*,* using phase contrast imaging

To determine the effect of prepared insulin-iron nanoparticles on in vitro wound healing HEKa cells were cultured in 60 mm plates along with DMEM-F12 media (FBS-free medium). The cells were kept in an incubator at 37 ^˚^C and 5% CO_2_ level until reaching 80–85% confluency. Once the plates became confluent, the cell scratch method was used to assess the healing response. In this method, wounds were created using a sharp object with a sterile 200 µl tip and subsequently treated with varying concentrations of IFe(II)NPs, insulin, FeSO_4_ salt solution, and a combination of insulin and FeSO_4_ salt (I + Fe). Time-lapse imaging was conducted to monitor changes in wound diameter, and the alterations in wound width were measured after 8 h, 16 h, and 32 h, respectively. We randomly measured wound width at different positions for each scratch made in an individual well plate and took the mean of those independent readings of wound diameter to calculate the percentage change in wound diameter for the normal wounds [5].

### Antioxidant activity against H_2_O_2_-induced cytotoxicity

To assess antioxidant activity against H_2_O_2_-induced cytotoxicity, HEKa cells were initially seeded in 96 well plates and maintained at 37 °C and 5% CO_2_ for 24 h in DMEM medium supplemented with 10% FBS until reaching confluency of 85–90%. The cells were then divided into two sets for different treatments. In the first test, cells were treated with varying concentrations of H_2_O_2_ (0, 50, 100, 200, 400, 800, 1200, 1600, and 2000 μM) in DMEM for a brief period of 1 h. Subsequently, the cells were washed twice with PBS to eliminate the H_2_O_2_. The cells in the second set were incubated with varying concentrations of H_2_O_2_ (0, 50, 100, 200, 400, 800, 1200, 1600, 2000 μM) for 1 h followed by the change of media and addition of IFe(II)NPs for 12 h. Following these treatments, an MTT assay was employed to determine the impact on cell viability after treatment with H_2_O_2_ and to investigate the potential antioxidant effect of the synthesized nanoparticles on the cells. The culture media was discarded, replaced with fresh media containing MTT, and left to incubate for 3 h. Followed by the addition of DMSO, the absorbance at 570 nm was measured for evaluation of the antioxidant properties of IFe(II) NPs in mitigating H_2_O_2_-induced cytotoxicity [50–52].

### Detection of ROS generation by flowcytometry

Flowcytometric analysis was conducted to determine the ROS generation by using 2’,7’dichlorofuorescin diacetate (DCF-DA). For this, HEKa cells were cultured in the presence of DMEM media until reaching 70% confluency. Subsequently, the cells were treated with varying concentrations of H_2_O_2_ (0, 200, 1000, 4000 μM) for a period of 1 h. Thereafter, the cells were treated with IFe(II)NPs (30 µM) for an additional 12 h. To assess ROS levels, cells were detached using trypsin–EDTA and suspended in PBS (0.5 ml) with 10 μM DCF-DA. The resulting mixture was then incubated for 10 min at 37 °C before undergoing flow cytometry analysis. A total of 10,000 cells were analyzed from each sample [53–55].

### Determination of combination index (CI) for iron (II) and insulin

The combination index (CI) is a quantitative measure that is used to determine the effect of two different drugs in combination with each other. This interaction can give either a synergistic or antagonistic effect, and the degree or level of synergism or antagonism is calculated by finding out the drug combination index. A CI < 1 value value indicates a synergistic effect, meaning that two drugs when administered together, enhance each other’s activity. On the other side a CI value exceeding 1 (CI > 1) exhibits the antagonistic effect of drugs, indicating that one of the drugs is responsible for inhibiting the activity of the other drug. The CI = 1 value suggests that neither drug interferes with the other.

The CI was computed by evaluating the cell viability of HEKa cells at varying.

concentrations of iron (II) and insulin, using Eq. (2).

3 $$ CI = \frac{{(D)_{1} }}{{\left( {Dx} \right)_{1} }} + \frac{{\left( D \right)_{2} }}{{\left( {Dx} \right)_{2} }} $$

where,

4 $$Dx=Dm {[fa/fu]}^{1/m}$$

Here, (D)_1_ and (D)_2_ denote the concentration of iron salt and insulin, respectively. The concentrations (Dx)_1_ and (Dx)_2_, indicative of the median effective doses for the individual drugs, are determined through Eq. (3), where the affected and unaffected cell fractions in the median dose are denoted by fa and fu and are equal to 10^(y–intercept)/m^, where m represents the slope median in the median effect plot of log (D) vs. log (fa/fu) [56].

### Statistical analysis

All the data presented here is expressed as the mean ± standard deviation (SD) and is derived from at least three independent experiments. Statistical data analysis was performed using one-way ANOVA in MS Excel and the measurements in which the *p*-values were ≤ 0.05 were considered to be statistically significant.

## Results and discussions

### Structure and composition of metal insulin clusters (IFe(II)NPs)

**Fig. 1:**
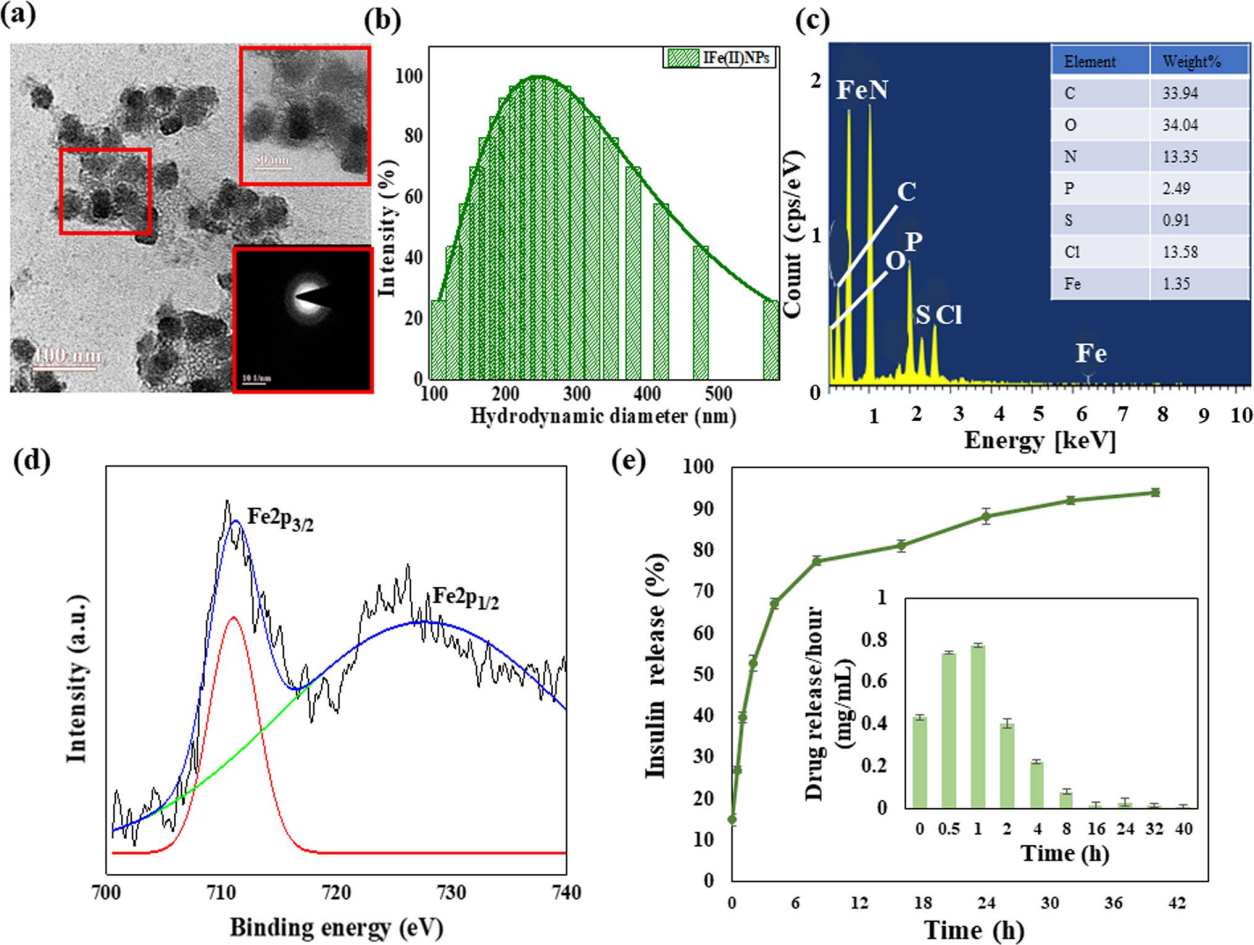
Morphological Characterization of IFe(II)NPs and release kinetic studies **a** HR-TEM images on a scale of 100 nm **b** DLS showing the hydrodynamic size of the synthesized Insulin-Iron nanoparticles (IFe(II)NPs) as 242.42 nm **c** EDS showing that iron is present in IFe(II)NPs as 0.35% **d** The XPS plot shoeing the elemental and chemical composition of the synthesized material **e** The plot displays the release kinetic studies conducted to determine the percentage drug release from IFe(II)NPs and the inset of the graph represents the drug release per h in mg/ml

The morphological characteristics of synthesized formulations were investigated using High-Resolution Transmission Electron Microscope (HRTEM). The formulations were found to be spherical in shape, and the size of the nanoparticles came out to be 28.6 ± 5.2 nm, as shown in Fig. 1a. Additionally, the Selected Area Diffraction ( SAED) pattern, shown in the inset of Fig. 1a confirms the partially amorphous nature of the synthesized clusters. The nanoparticles are evenly distributed within the protein matrix. The FE-SEM analysis was done to study the surface morphology as shown in Fig. S1a. The dynamic light scattering (DLS) technique was used to find the hydrodynamic size of IFe(II)NPs, which was determined to be approximately 242.42 nm, as shown in Fig. 1b, and the stability of nanoparticles after 3 months was determined using DLS which confirms the particle size to be 256 nm as shown in Fig. S1b. To determine the different elements present in the samples, energy dispersive X-ray spectroscopy was used, which confirmed the presence of C, O, P, S, Cl, and Fe, see Fig. 1c. Fe was found to be evenly distributed in the sample, constituting approximately 1.35% of the overall composition, as indicated in the inset of Fig. 1c.

### XPS analysis

X-ray photoelectron spectroscopy (XPS) was carried out for the prepared formulation IFe(II)NPs, and the corresponding spectra are illustrated in Fig. 1d. Within this figure, two peaks were observed: Fe 2*p*_3/2_ and Fe 2*p*_1/2_. Notably, the Fe 2*p*_3/2_ peak is observed to be narrower and stronger than the Fe 2*p*_1/2_ peak. The binding energies for these peaks, Fe 2*p*_3/2_ and Fe 2*p*_1/2_, are 711.08 eV and 727.45 eV, respectively. The presence of Fe(II) in IFe(II)NPs is confirmed by these observed peaks [41].

### Drug loading and release kinetics

To assess the drug loading capacity and release kinetics of IFe(II)NPs, experiments were conducted under physiological conditions, specifically at pH 7.4 and 37 °C. The encapsulation efficiency of insulin protein within IFe(II)NPs was quantified and found to be 88.54 ± 0.038%. For the analysis of release kinetics, particles were enclosed in a dialysis membrane with a drug concentration of 3.47 mg/ml. Samples were systematically collected, and their absorbance at 595 nm was measured. Initially, there was a rapid release of the drug within the first 8 h, succeeded by a sustained release extending over approximately 30 h. At the culmination of the 40 h timeframe, an impressive 93.90 ± 0.90% of the insulin had been released, as depicted in Fig. 1e. This release accounts for 2.88 mg/ml by the 40-h mark, as illustrated in the inset of Fig. 1e. This outcome underscores the efficacy of IFe(II)NP as a robust drug delivery system.

### In silico studies to determine the interactions between Fe(II) and insulin protein

**Fig. 2:**
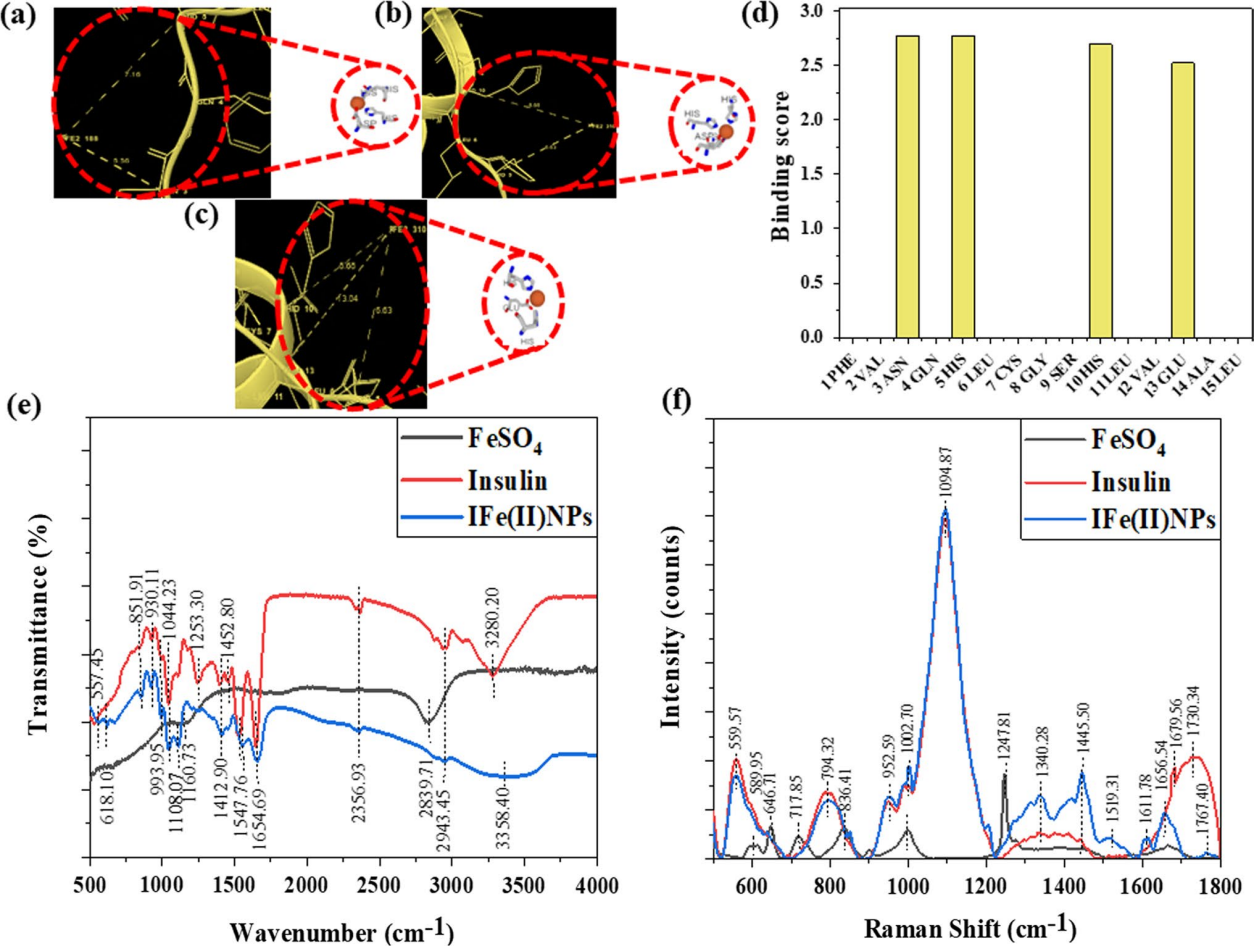
The Fig. shows the in silico studies using MIB software for finding the binding sites of Fe(II) on insulin and further confirming the interactions between two by FTIR and Raman spectroscopy **a** Metal ion binding residues showing binding of Fe(II) with amino acids on the chain B of the Insulin protein with amino acids 3 ASN and 5 HIS, and the distance between the metal ion and amino acids is 5.56 Å and 7.16 Å, respectively **b** Binding of Fe(II) with amino acids on the chain B of the Insulin protein with 5 HIS and 10 HIS, and the distance measured is 6.63 Å and 8.68 Å respectively **c** Binding of Fe(II) with amino acids on the chain B of the Insulin protein with 5 HIS, 10 HIS, and 13 GLU the distance was found to be 10.73 Å, 4.48 Å and 6.58 Å respectively **d** The plot depicts the binding potential of each amino acid with Fe(II) **e** FTIR revealing the interaction between protein Insulin and iron after the formation of nanoparticles **f** RAMAN providing us with the structural fingerprint by which different molecules can be defined

The MIB (Metal Ion Binding) online docking server was utilized to identify potential binding sites on the insulin protein for the transition metal Fe(II). The interaction between the metal and protein is heavily reliant on the amino acid sequence and structure within the protein. Initially, the human insulin sequence was extracted from the Protein Data Bank under the ID 4EWW. Subsequently, this ID was input into the MIB tool to facilitate the docking of Fe(II) ions with insulin’s chains A & B. This process involved assigning a binding score to each residue within the insulin protein, helping identify specific binding sites based on a predefined threshold. Analysis of the data revealed an absence of binding sites for Fe(II) on chain A. However, chain B exhibited three distinct binding sites for Fe(II) ions. Further investigation was conducted using the Maestro software to calculate the distances between the metal ion and its corresponding binding amino acids. For Fe(II), three distinct grooves were identified. In groove 1, the metal ion bound with amino acids ASN at position 3 and HIS at position 5, with respective distances of 5.56 Å and 7.16 Å from the metal ion. Groove 2 showed binding with HIS at positions 5 and 10, with measured distances of 6.63 Å and 8.68 Å, respectively. Lastly, groove 3 demonstrated binding with HIS at positions 5 and 10, along with GLU at position 13, at distances of 10.73 Å, 4.48 Å, and 6.58 Å, respectively, as illustrated in Fig. 2a–c. Additionally, the software provided binding scores for various amino acids, aiding in the selection of the most suitable binding sites, as depicted in Fig. 2d. This comprehensive analysis facilitated a detailed understanding of the Fe(II) binding sites within the insulin protein structure.

### Study of the interactions between insulin and FeSO_4_ after IFe(II)NPs formation using FTIR and Raman spectra

FTIR was done for insulin along with both FeSO4 and IFe(II)NPs to find out the interactions between protein and metal ions. A peak appeared at 557.45 cm^−1^ in IFe(II)NPs and 559.32 cm^−1^ in FeSO_4_ but was absent in insulin, indicating the Fe–O stretching and bending [57]. Another peak of aromatic C-S stretch was observed at 618.10 cm^−1^ in insulin and shifted to 667.03 cm^−1^ in IFe(II)NPs [12]. Then, a peak appears at 851.91 cm^−1^ in both insulin and IFe(II)NPs indicating the NH_2_ stretch [12]. Further, a peak at 930.11 cm-1 indicates the presence of the C–O–C bond in insulin and in IFe(II)NPs [4]. A peak comes at 993.95 cm^−1^ in both insulin and IFe(II)NPs indicating C-O stretching but absent in FeSO_4_ [12]. For the C-N stretch, the peak comes at 1044.23 cm^−1^ in insulin and IFe(II)NPs [13]. For the S = O stretch, a peak comes at 1161.80 cm^−1^ in FeSO_4_, 1108.07, and 1160.73 cm^−1^ in insulin and IFe(II)NPs [12]. The peak at 1253.30 cm^−1^ indicates an amide III bond in insulin as well as IFe(II)NPs [5]. Amide II bond peaks at 1412.90 cm^−1^ in insulin and 1419.24 cm^−1^, 1452.80 cm^−1^ in IFe(II)NPs, indicating their formation [4]. C–C stretch was there at 1547.76 cm^−1^ in insulin and IFe(II)NPs [4]. A peak at 1654.69 cm^−1^ indicates Amide I in insulin and IFe(II)NPs [4]. Nitrile stretch was visible at 2356.93 cm^−1^ in insulin and IFe(II)NPs [13]. O–H stretch was observed at 2839.71 cm^−1^ in FeSO_4_ and shifted to a higher wavenumber 2943.45 cm^−1^ in insulin and IFe(II)NPs [13]. Amine N–H stretch was also there in both insulin and IFe(II)NPs but at different wave numbers that are 3358.40 cm^−1^ in IFe(II)NPs, and 3280.20 cm^−1^ in insulin [58] as demonstrated in Fig. 2e and Additional file 1: Fig. S2.

To study the structural changes in the protein level, Raman was performed. S–S peak is observed at 589.95 cm^−1^ for FeSO_4_ and at 559.57 cm^−1^ in insulin and IFe(II)NPs [59]. The antisymmetric bending (SO_4_) peak comes out to be at 646.71 cm^−1^ and 717.85 cm^−1^ for FeSO_4_ and is absent in the case of insulin and IFe(II)NPs [60]. Further, the peak at 836.41 cm^−1^ is for the O-C-N bend observed in FeSO_4_ and shifted to a lower wavenumber 794.32 cm^−1^ in insulin and IFe(II)NPs [60]. The peak responsible for C–O–C at 952.59 cm^−1^ was seen in the case of insulin and IFe(II)NPs but was absent in FeSO_4_ [13]. At 997.32 cm^−1^, there was a peak for symmetric stretching (SO_4_) bond, which is seen only in the case of FeSO_4_ [61]. Similarly, two peak positions were observed, 1002.70 cm^−1^ and 1094.87 cm^−1^, in insulin and IFe(II)NPs corresponding to the C-N stretch [62]. An antisymmetric stretching (SO_4_) at 1247.81 cm^−1^ in FeSO_4_ was inferred [63]. Then, a peak at 1340.28 cm^−1^ and 1337.30 cm^−1^ for insulin and IFe(II)NPs, respectively, corresponds to Amide III (random coils) [13]. 1445.50 cm^−1^ and 1519.31 cm^−1^ peak position confirms the presence of amide II bond in IFe(II)NPs [60], and for amide I bond, the peak position is 1656.54 cm^−1^, 1679.56 cm^−1^ in insulin and 1656.74 cm^−1^, 1611.78 cm^−1^ in IFe(II)NPs [64]. Lastly, C = O (stretch) bond position at 1730.34 cm^−1^ is observed in insulin, which shifts to 1767.40 cm^−1^ in IFe(II)NPs [60], as shown in Fig. 2f. A comparative data of FTIR and Raman spectra are shown in Table 1.

**Table 1:** The table gives the comparative values of the wavenumbers obtained from the FTIR (in the range of 400–4000 cm^−1^) and Raman spectra (500–1800 cm^−1^) respectively of FeSO_4_, Insulin, and IFe(II)NPs indicating changes in different functional groups present, thus coins the interaction among insulin protein and FeSO_4_ leading to the formation of IFe(II)NPs

Functional groups	FeSO_4_	Insulin	IFe(II)NPs	Reference	Functional group	FeSO_4_	Insulin	IFe(II)NPs	Reference
Fe–O bond	559.32	–	557.45	[57]	S–S stretch	589.95	559.57	559.57	[59]
C–S stretch	–	618.10	667.03	[12]	Antisymmetric bending (SO_4_)	646.71 717.85	–	–	[60]
NH_2_ stretch	–	851.91	851.91	[12]	O–C–N Bend	836.41	794.32	794.32	[60]
C–O–C bond	–	930.11	930.11	[4]	C–O–C	–	952.59	952.59	[13]
C–O stretch	–	993.95	993.95	[12]	Symmetric stretching (SO_4_)	997.32	–	–	[61]
C–N stretch	–	1044.23	1044.23	[13]	C–N Stretch	–	1002.70 1094.87	1002.70 1094.87	[62]
S=O stretch	1161.80	1108.07 1160.73	1108.07 1160.73	[12]					
Amide III		1253.30	1253.30	[5]					
Amide II	–	1412.90	1412.90 1452.80	[4]	Antisymmetric stretching (SO_4_)	1247.81	–	–	[63]
C–C stretch	–	1547.76	1547.76	[4]	Amide III (Random coils)	–	1340.28	1337.30	[13]
Amide I	–	1654.69	1654.69	[4]	Amide II	–	1443.01 1519.31	1445.50 1519.31	[60]
Nitrile stretch	–	2356.93	2356.93	[13]	Amide I	–	1656.54 1679.56	1656.74 1611.78	[64]
O–H stretch	2839.71	2943.45	2943.45	[13]	C=O Stretch	–	1730.34	1767.40	[60]
Amine N–H stretch	–	3280.20	3358.40	[58]					

### In vitro cell line studies

#### Cell viability studies

**Fig. 3:**
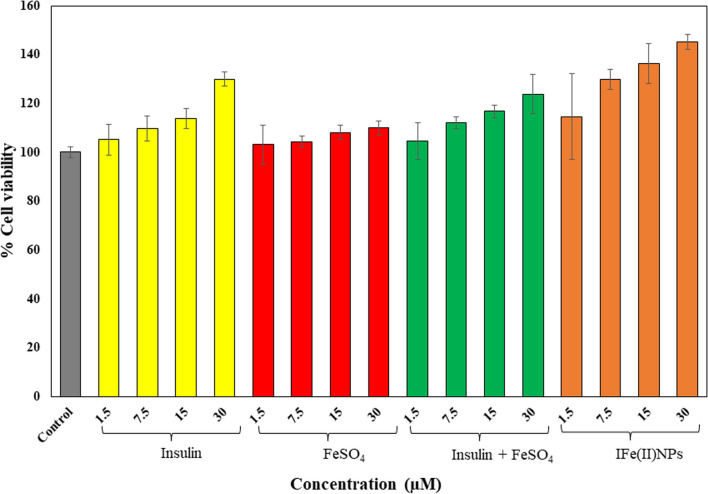
Shows the in vitro* studies* for determining the cell viability. The MTT assay determined the growth rate of HEKa cells. The data shows the treatment of HEKa cells with Insulin, FeSO_4,_ Insulin + FeSO_4,_ and IFe(II)NPs at concentrations 1.5, 7.5, 15, and 30 µM for each sample. The data were plotted as the mean of three independent experiments

The MTT result for cell viability is highly dependent on the mitochondrial activity of cells. To this end, we calculated % cell viability for varying concentrations (1.5, 7.5, 15, and 30 μM) of insulin, FeSO_4_, a mixture of insulin and FeSO_4_, and IFe(II)NPs, see Fig. 3 for a graphical presentation. The whole experiment was repeated thrice, and averages of all those readings were calculated and plotted along with the error bars based on standard deviation. For control cells, cell viability is taken as 100%.

For insulin, cell viability showed a steady increase: 105.192 ± 6.25% for 1.5 μM, 109.67 ± 5.05% for 7.5 μM, 113.86 ± 4.02% for 15 μM, and 129.88 ± 2.89% for 30 μM. FeSO_4_ treated cells displayed percentage viability as 103.12 ± 8.00% for 1.5 μM, 104.36 ± 2.31% for 7.5 μM, 108.14 ± 2.96% for 15 μM, 109.91 ± 2.88% for 30 μM. The combined treatment with a combination of insulin and FeSO_4_ showed a synergistic growth-promoting effect of insulin-iron NPs on cell viability: 104.42 ± 7.49% for 1.5 μM, 112.12 ± 2.33% for 7.5 μM, 116.72 ± 2.61% for 15 μM, 123.77 ± 7.93% for 30 μM. However, IFe(II)NPs treated cells displayed the highest across all samples: 114.60 ± 17.55% for 1.5 μM, 129.79 ± 4.16% for 7.5 μM, 136.28 ± 8.20% for 15 μM and 145.133 ± 2.96% for 30 μM. The comparative data is shown in Table S1, suggesting that insulin-iron nanoparticles’ are acting as cell growth-promoting particles hence, their potential for wound healing should be further explored. To assess the statistical significance of the data p values calculated for % change in cell viability after treatment with varying concentrations of samples and are given in Additional file 1: Table S2. The data is considered to be statistically significant since all values of p are < 0.05.

### Fluorescence quantum yield

**Fig. 4:**
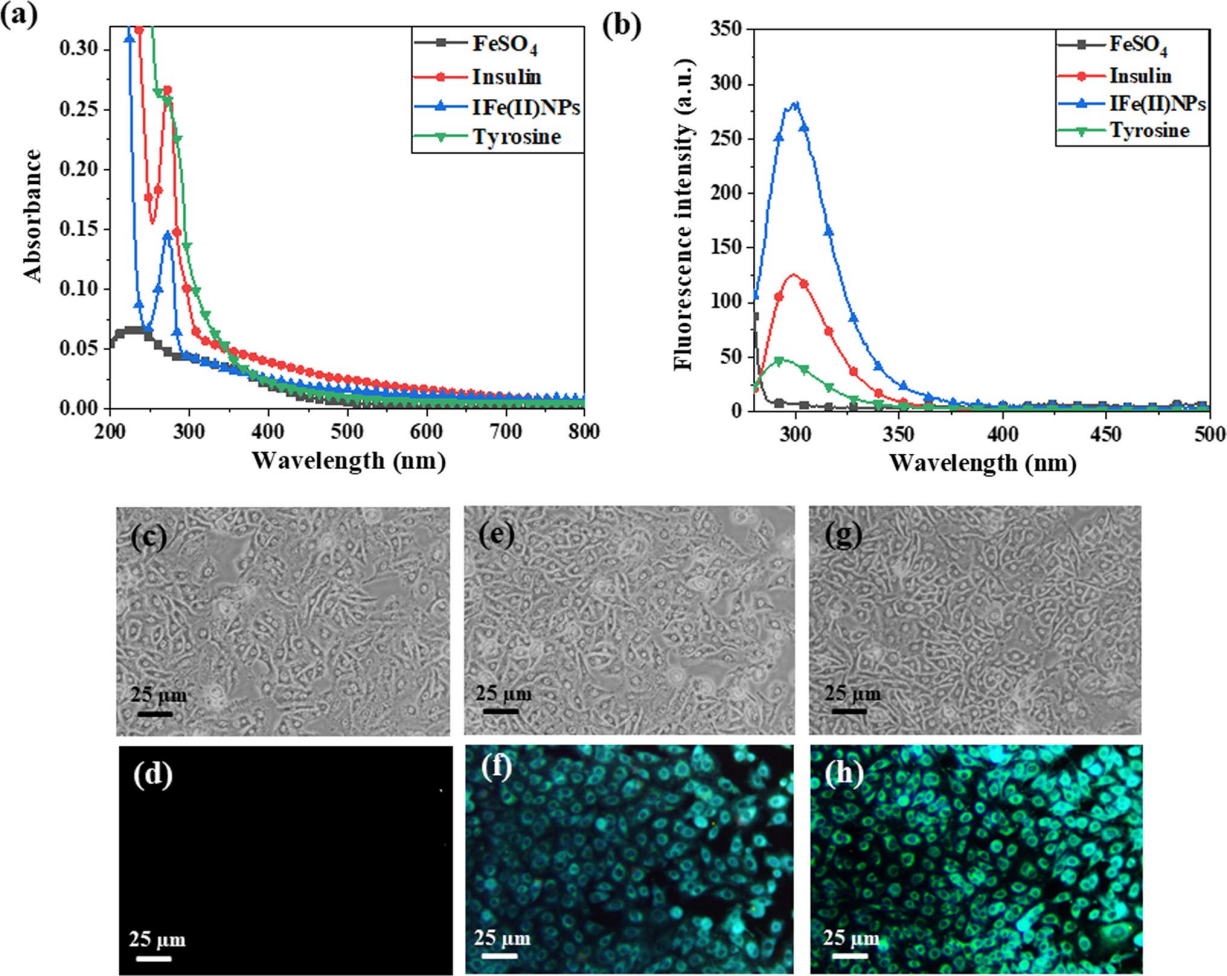
Provides information on the spectroscopic changes after the interaction between insulin and iron salt and further confirms the fluorescent properties by performing bioimaging on HEKa cells **a** Shows the excitation peak of insulin was shown at 272 nm in absorption spectra, and after the formation of IFe(II)NPs, the maxima were obtained at 272 nm in absorption spectra **b** It shows the fluorescence spectra after exciting Insulin, and IFe(II)NPs at 272 nm and an emission spectrum was obtained which reveals the maximum fluorescence intensity at ~ 300 nm with emission range of 280–360 nm. Displays the in vitro imaging on HEKa cell line using IFe(II)NPs **c** shows cells without any treatment with the synthesized IFe(II)NPs under white light **d** cells without treatment under violet light **e** cells treated with 30 µM of IFe(II)NPs under white light **f** cells treated with 30 µM of IFe(II)NPs under violet light both in absence of magnet **g** cells treated with 30 µM of IFe(II)NPs under white light **h** cells treated with 30 µM of IFe(II)NPs under violet light both in presence of a magnet

Following the procedure outlined in Sect. 2. 9, the synthesized IFe(II)NPs samples were characterized for their spectroscopic properties. Referring to Fig. 4a, for insulin without added metal salts, a sharp peak was observed at 272.03 nm wavelength with an absorbance value of 0.246 units. In contrast, after incubating the insulin with metal salts for 48 h, an absorbance value of 0.136 was obtained for nanoparticles that peaked at 272.9 nm. The absorption peak is also compared with that of a tyrosine as it was selected as a standard (Fig. 4a).

For a relative comparison of the quantum yield, before and after incubation of insulin with an iron salt, the fluorescence intensity value of insulin, FeSO_4_, IFe(II)NPs, and tyrosine (standard) are measured and presented in Fig. 4b. At an excitation wavelength of 272 nm, the emission spectra ranging from 280 to 360 nm revealed a maxima around 300 nm with an intensity of 118.15 a.u. for insulin. Monitoring the spectra from 200 to 800 nm for IFe(II)NPs, an intensity of 280.52 a.u. was observed, still around a wavelength of 300 nm, as shown in Fig. 4b. This is much higher than for the salt solution and insulin alone. Using tyrosine (with a known quantum yield), the quantum yield of insulin was determined to be s 0.179, while that that of IFe(II)NPs is 0.632, This result highlights the potential of IFe(II)NPs as a promising bioimaging agent. The calculated % change in fluorescence was calculated as 55.93.

### Fluorescence microscope bioimaging

To confirm the broad application of IFe(II)NPs for cellular imaging, bioimaging was done on the HEKa cell line. The cells were incubated initially for a time duration of 8 h with IFe(II)NPs having a concentration of 30 µM. Then the cells were fixed using a 2% formaldehyde solution, and after that, the cells were observed under white light and violet light. The cells showed greenish-blue fluorescence because of the binding of insulin-iron nanoparticles with the insulin receptors on the cell wall as they illuminate them and make them appear fluorescent. Also, the images were taken, both in the absence and presence of a magnet as shown in Fig. 4c-h, and the intensity of fluorescence increases in the presence of magnetic field. This study infers that nanoparticles emit bright fluorescence and can be used for bioimaging.

### Migration assay

**Fig. 5:**
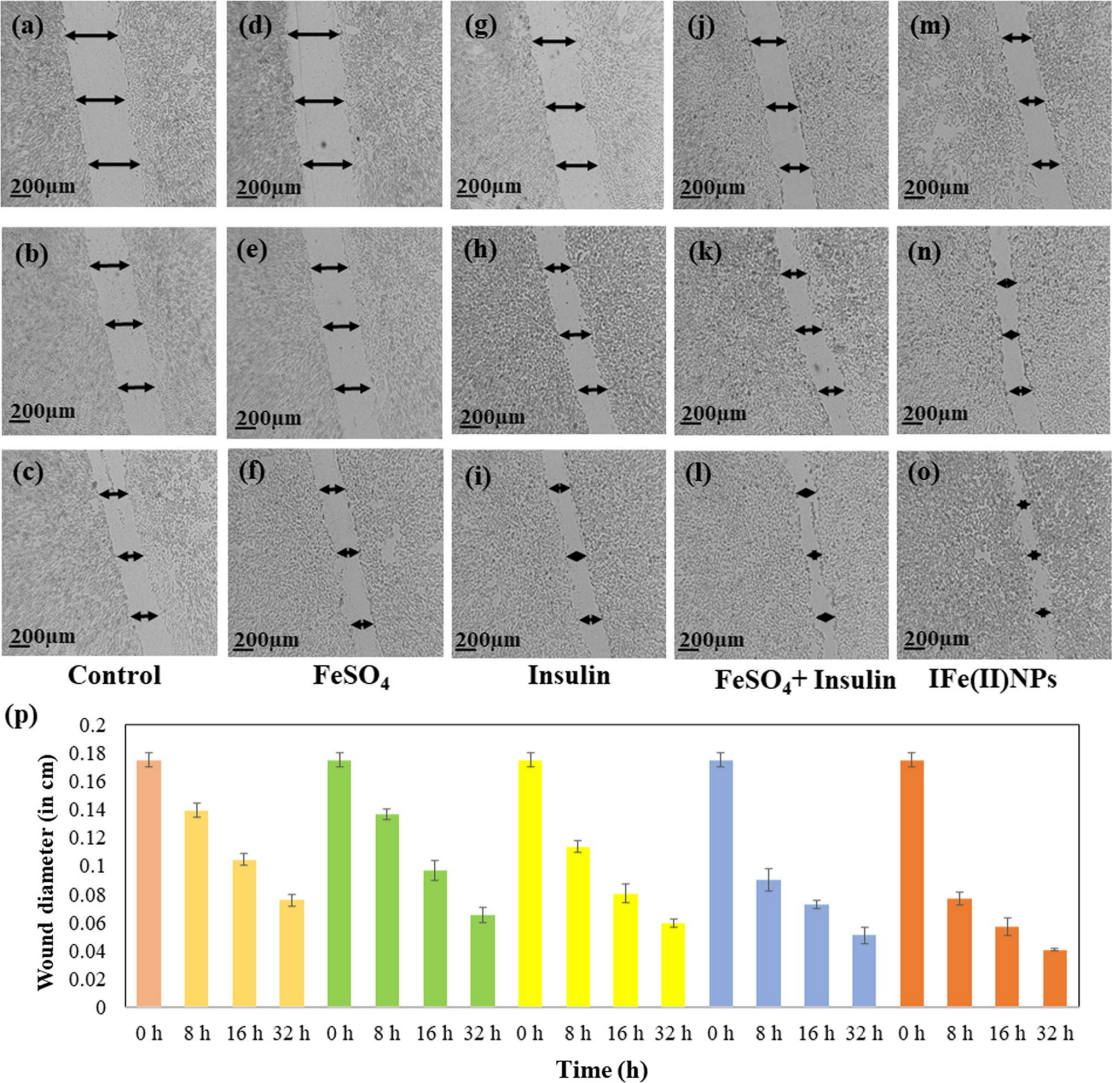
The Fig. shows the in vitro wound recovery using IFe(II)NPs. The synthesized Insulin-iron nanoparticles displayed better wound healing in HEKa than alone insulin, FeSO_4_ salt solution, and the mixture of insulin and FeSO_4._ The HEKa cells were incubated with a fixed concentration of 30 µM for each sample. Control cells without any sample treatment **a** 8 h **b** 16 h **c** 32 h. Cells treated with FeSO_4_ salt solution **d** 8 h **e** 16 h **f** 32 h. Cells treated with Insulin **g** 8 h **h** 16 h **i** 32 h. Cells treated with FeSO_4_ + insulin **j** 8 h **k** 16 h **l** 32 h. Cells treated with the synthesized IFe(II)NPs **m** 8 h **n** 16 h **o** 32 h. **p** Plot showing the comparison of the change in wound diameter post-treatment with Insulin, FeSO_4_ salt solution, the mixture of insulin and FeSO_4_ and IFe(II)NPs including control after 8 h, 16 h, and 32 h

The in vitro wound recovery data, displayed in Fig. 5 demonstrate that IFe(II)NPs exhibit a significantly higher degree of cell migration compared to untreated and treated controls, including FeSO_4_, insulin, and insulin + FeSO_4_. There is an increase in the extent of both cell division and migration with the increase in time at a fixed concentration of 15 μM. To quantify the changes in wound diameter, we measured wound width at three different positions for each scratch made in an individual well plate. The mean of those independent readings was used to calculate the percentage change in wound diameter. In comparison to the control scratch diameter, IFe(II)NPs-treated cells showed the percentage of a gap left between the scratched wound after 8 h, 16 h, and 32 h as 40.93 ± 0.58%, 38.92 ± 0.58%, and 31.76 ± 0.26%, respectively. Similarly, cells treated with FeSO_4_, Insulin, and Insulin + FeSO_4_, showed significant migration compared to the control, as reflected in the percentage gap. For e cells treated with Insulin + FeSO_4,_ this gap left in scratch was 62.26 ± 0.90% after 8 h, 59.09 ± 0.20% after 16 h, and 55.68 ± 0.57% after 32 h. For cells incubated with insulin, the corresponding numbers were 75.26 ± 0.40%, 68.46 ± 0.66%, and 57.64 ± 0.23%, respectively Similarly, the cells treated with FeSO_4_ showed the gap left as 87.42 ± 0.37%, 84.09 ± 0.66%, and 74.40 ± 0.51% after the time duration of 8 h, 16 h and 32 h, respectively.

The enhancement of migration ability with varying times, as noted above, confirms the wound-healing effect of insulin-iron nanoparticles, as illustrated in Fig. 5a–o. The post-treatment wound diameter with insulin, FeSO_4_ salt solution, and the mixture of insulin and FeSO_4_ and IFe(II)NPs including control after 8 h, 16 h, and 32 h. is shown in Fig. 5p. To assess the statistical significance of the data p values calculated for % variation in wound diameter are listed in Additional file 1: Table S3. The data is considered to be statistically significant since all values of *p* are < 0.05.

### IFe(II)NPs mediated protection against external oxidative stress

**Fig. 6:**
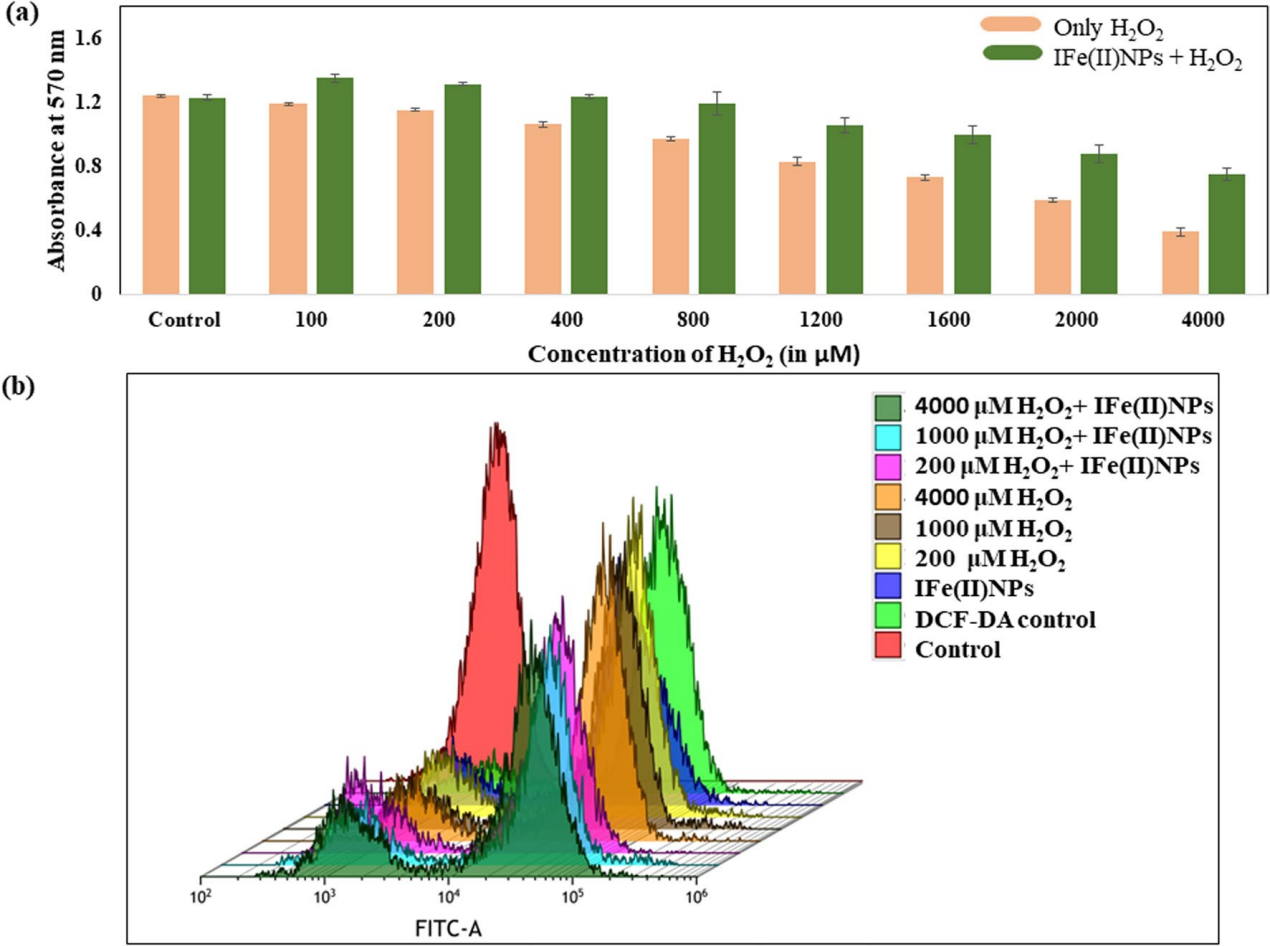
The Fig. depicts the ROS scavenging activity of synthesized nanoparticles **a** It shows the effect of increasing H_2_O_2_ concentration against the cell viability of HEKa cells in the presence and absence of IFe(II)NPs **b** It depicts the flow cytometry analysis of ROS scavenging in presence of IFe(II)NPs

Enhancement of oxidative stress in the tissue microenvironment is a characteristic feature of proinflammatory signaling which in turn inhibits cell growth and migration. The H_2_O_2_ is found to be toxic to the cells (HEKa) at a concentration of 400 μM or higher and eventually causes a decrease in the cell viability with increasing concentration from 0 to 4000 μM. On the other side, the cells treated with the fixed concentration of IFe(II)NPs that is 30 μM show ROS scavenging activity against the H_2_O_2_-induced cytotoxic behavior by overcoming the toxic impact of H_2_O_2_ even at a high concentration of 4000 μM. In cells initially treated with 100, 200, 400, 800, and 1200 μM of H_2_O_2_ there is a percentage ROS scavenging of 14.02 ± 2.90%, 14.10 ± 0.76%, 14.84 ± 1.40%, 18.38 ± 7.34% and 19.27 ± 4.56% respectively after treatment with 30 μM IFe(II)NPs. The ROS scavenging is even higher in cells initially treated with 1600, 2000, and 4000 μM of H_2_O_2_ and comes out to be 22.23 ± 5.75%, 23.97 ± 5.64%, and 29.24 ± 3.60% respectively which makes them potent ROS scavenging formulations as shown in Fig. 6a.

### Protection of intercellular oxidative stress by IFe(II)NPs

To monitor the intercellular ROS scavenging activity of IFe(II)NPs, flow cytometry was used. The conversion of DCF-DA into cell impermeable green fluorescent products is induced by intracellular ROS that can be measured by flow cytometry. Initially, the flow cytometry was done for control cells and DCFDA control cells. Then in comparison to the DCFDA control, other samples; 200 µM H_2_O_2_, 1000 µM H_2_O_2_, 4000 µM H_2_O_2,_ only IFe(II)NPs, 200 µM H_2_O_2_ + IFe(II)NPs, 1000 µM H_2_O_2_ + IFe(II)NPs and 4000 µM H_2_O_2_ + IFe(II)NPs were analyzed. As a result, it was observed that the increase in ROS percentage after treatment with 200, 1000, and 4000 µM H_2_O_2_ is 22.81%, 38.56%, and 47.05% respectively. There are a reduction of 26.83% and 19.41% in cells treated with 1000, and 4000 µM H_2_O_2_ + IFe(II)NPs (30 μM) respectively and an even higher reduction was observed in cells treated with 200 µM H_2_O_2_ + IFe(II)NPs (30 μM) which is 5.49% less than the DCFDA control itself indicating a strong ROS scavenging potential of synthesized IFe(II)NPs as shown in Fig. 6b.

### Combination index of iron and insulin

To calculate the combination index, Dm was calculated using m and y from Additional file 1: Fig. S3a for iron and Additional file 1: Fig. S3b for insulin, respectively. From the values computed using CI, see Table 2, it became evident that iron sulfate and insulin exhibit a synergistic effect. The calculated values for iron sulfate and insulin were found to be less than one, indicating that both enhance each other’s activity by working together and exhibit potential synergistic effects. Similar results were obtained from the in vitro tests performed to determine cell viability and those performed for the cell migration assay for efficient wound healing activity.

**Table 2:** The table provides the data of the Combination Index (CI) calculated from the cell viability data by varying concentrations of iron and insulin in combination to check if the two drugs exhibit synergistic or antagonistic effect

Concentration of Fe salt and Insulin	(Dx)1 (Fe salt) = Dm[fa/fu]^1/m^	(Dx)1 (Insulin) = Dm[fa/fu]^1/m^	CI = (D)1/(Dx)1 + (D)2/(Dx)2
1.5 µM	52473.9	5714.1	0.000291
7.5 µM	534.199	3489.39	0.016189
15 µM	87.677	588.616	0.196566
30 µM	62.048	150.719	0.682536

The values come out to be less than 1, thus indicates the synergistic effect of iron and insulin

## Conclusion

Wound healing poses significant challenges, especially in severe conditions, necessitating careful monitoring to track the dynamic changes occurring at the wound site. Delays in this process often result from inflammation and the presence of external reactive oxygen species (ROS). Hence, the development of innovative formulations becomes paramount, not only for enhancing wound healing but also for enabling bioimaging, targeted drug delivery, and ROS scavenging capabilities. This study successfully synthesized IFe(II)NPs, confirmed through a series of comprehensive analyses.

Moreover, the insulin-iron nanoparticles exhibited target specificity by utilizing the widely present insulin protein receptors in the human body. Their high fluorescence emission, particularly intensified in the presence of a magnetic field, makes them promising for bioimaging applications. The interaction between this formulation and cell receptors can be effectively detected through fluorescence imaging. Notably, these nanoparticles displayed ROS scavenging activity, evident from the observed decrease in fluorescence intensity analyzed via flow cytometry. Further, we have compared these formulations with previously synthesized nanoformulations using insulin protein in wound healing and found that these formulations have much potential in increasing cell viability and cell migration even at half the concentration used previously. These findings hold promise for preclinical investigations, suggesting potential applications in animal models to evaluate safety and efficacy before clinical trials. Moving towards preclinical and clinical studies, these nanoparticles could be explored for their therapeutic potential in human subjects, offering possibilities for addressing complex wounds and exploring their diagnostic and therapeutic roles in clinical settings. The multifunctional nature of these nanoparticles suggests a wide range of preclinical and clinical applications, urging further exploration to harness their full potential in biomedical and clinical contexts.

### Supplementary Information

**Additional file 1: Figure S1.** Figure shows (**a**) FE-SEM image to study the surface morphology of synthesized (**b**) DLS for confirming the stability of the synthesized nano-formulation after 3 months (92 days).** Figure S2.** FTIR spectra to confirm the interaction between protein Insulin and iron after formation of nanoparticles (500–1600 cm^-1^).** Figure S3.** Median plots of (**a**) FeSO_4_ salt (**b**) Insulin for finding the y-intercept and m values to calculate Dm to determine the combination index of iron and insulin.** Table S1.** The table shows the % variation in mitochondrial reductase activity in MTT assay for determining cellular metabolism rate using HEKa cells. The cells were treated with varying concentrations; 1.5 µM, 7.5 µM, 15 µM, and 30 µM of Insulin, FeSO_4_, Insulin + FeSO_4_, and IFe(II)NPs for a duration of 24 h. The data was plotted as mean value ± SD of three independent experiments.** Table S2.** It shows the p values calculated for % change in cell viability after treatment with varying concentrations of Insulin, FeSO_4_, Insulin + FeSO_4_ and IFe(II)NPs. The data is considered to be statistically significant when* p* < 0.05.** Table S3.** It shows the p values calculated for % variation in wound diameter after treatment with 15 µM of Insulin, FeSO**4**, Insulin + FeSO**4**, and IFe(II)NPs. The data is considered to be statistically significant when* p* < 0.05.

## Data Availability

The authors declare that the data supporting the findings of this study are available within the paper and its Additional file 1. Should any data files be needed in any other format, they are available from the corresponding author upon reasonable request.

## References

[1] Shang L, Dong S, Nienhaus GU (2011). Ultra-small fluorescent metal nanoclusters: synthesis and biological applications. Nano Today.

[2] Attri K, Sharda D, Chudasama BN, Mahajan R, Choudhury D (2023). A review on terpenes for treatment of gastric cancer: current status and nanotechnology-enabled future. RSC Sustain.

[3] Muhammed MAH, Pradeep T. Luminescent Quantum Clusters of Gold as Bio-Labels. In: Advanced Fluorescence Reporters in Chemistry and Biology. 2010. 10.1007/978-3-642-04701-5_11.

[4] Kaur P, Sharma AK, Nag D, Das A, Datta S, Ganguli A, Goel V, Rajput S, Chakrabarti G, Basu B, Choudhury D (2019). Novel nano-insulin formulation modulates cytokine secretion and remodeling to accelerate diabetic wound healing. Nanomedicine Nanotechnol Biol Med.

[5] Sharda D, Choudhury D (2023). Insulin–cobalt core–shell nanoparticles for receptor-targeted bioimaging and diabetic wound healing. RSC Adv.

[6] Teot L, Ohura N (2021). Challenges and management in wound care. Plast Reconstr Surg.

[7] Sharda D, Attri K, Choudhury D (2023). Future research directions of antimicrobial wound dressings. Antimicrob Dressings.

[8] Las Heras K, Igartua M, Santos-Vizcaino E, Hernandez RM (2020). Chronic wounds: current status, available strategies and emerging therapeutic solutions. J Control release.

[9] Wu H, Li F, Shao W, Gao J, Ling D (2019). Promoting angiogenesis in oxidative diabetic wound microenvironment using a nanozyme-reinforced self-protecting hydrogel. ACS Cent Sci.

[10] Wu YK, Cheng NC, Cheng CM (2019). Biofilms in chronic wounds: pathogenesis and diagnosis. Trends Biotechnol.

[11] Kaur P, Choudhury D (2019). Insulin promotes wound healing by inactivating NFkβ P50/P65 and activating protein and lipid biosynthesis and alternating pro/anti-inflammatory cytokines dynamics. Biomol Concepts.

[12] Kaur P, Choudhury D (2021). Functionality of receptor targeted zinc-insulin quantum clusters in skin tissue augmentation and bioimaging. J Drug Target.

[13] Sharda D, Attri K, Kaur P, Choudhury D (2021). Protection of lead-induced cytotoxicity using paramagnetic nickel-insulin quantum clusters. RSC Adv.

[14] Oryan A, Alemzadeh E. Effects of insulin on wound healing: A review of animal and human evidences. Life Sci. 2017;74:59–67. 10.1016/j.lfs.2017.02.015 10.1016/j.lfs.2017.02.015 28263805

[15] Sharda D, Ghosh S, Kaur P, Basu B, Choudhury D (2023). Chitosan-insulin nano-formulations as critical modulators of inflammatory cytokines and Nrf-2 pathway to accelerate burn wound healing. Discov Nano.

[16] Sharda D, Kaur P, Choudhury D (2023). Protein-modified nanomaterials: emerging trends in skin wound healing. Discov Nano.

[17] Kalashnikova I, Das S, Seal S (2015). Nanomaterials for wound healing: scope and advancement. Nanomedicine.

[18] Rajendran NK, Kumar SSD, Houreld NN, Abrahamse H (2018). A review on nanoparticle based treatment for wound healing. J Drug Deliv Sci Technol.

[19] Das S, Baker AB (2016). Biomaterials and nanotherapeutics for enhancing skin wound healing. Front Bioeng Biotechnol.

[20] Mohanty JS, Xavier PL, Chaudhari K, Bootharaju MS, Goswami N, Pal SK, Pradeep T (2012). Luminescent, bimetallic AuAg alloy quantum clusters in protein templates. Nanoscale.

[21] Xavier PL, Chaudhari K, Verma PK, Pal SK, Pradeep T (2010). Luminescent quantum clusters of gold in transferrin family protein, lactoferrin exhibiting FRET. Nanoscale.

[22] Tarhini M, Greige-Gerges H, Elaissari A (2017). Protein-based nanoparticles: from preparation to encapsulation of active molecules. Int J Pharm.

[23] Chaudhari K, Xavier PL, Pradeep T (2011). Understanding the evolution of luminescent gold quantum clusters in protein templates. ACS Nano.

[24] Alavi M, Hamblin R, Martinez F, Aghaie E, Khan H, Menezes IA, Micro and nanoformulations of insulin: new approaches. Nano Micro Biosyst. 2022;1:1–7. 10.22034/nmbj.2022.157284

[25] Chen PF, Liu CL, Lin WK, Chen KC, Chou PT, Chu SW, Betzig E, Patterson GH, Sougrat R, Lindwasser OW, Olenych S, Bonifacino JS, Davidson MW, Lippincott-Schwartz J, Hess HF, Huang B, Wang W, Bates M, Zhuang X, Chu W, Su TY, Oketani R, Huang YT, Wu HY, Yonemaru Y, Yamanaka M, Lee H, Zhuo GY, Lee MY, Kawata S, Fujita K (2015). Fluorescence depletion properties of insulin–gold nanoclusters. Biomed Opt Express.

[26] Ehterami A, Salehi M, Farzamfar S, Vaez A, Samadian H, Sahrapeyma H, Mirzaii M, Ghorbani S, Goodarzi A (2018). In vitro and in vivo study of PCL/COLL wound dressing loaded with insulin-chitosan nanoparticles on cutaneous wound healing in rats model. Int J Biol Macromol.

[27] Ribeiro MC, Correa VLR, da Silva FKL, Casas AA, Chagas AL, Oliveira LP, Miguel MP, Diniz DGA, Amaral AC, de Menezes LB (2020). Wound healing treatment using insulin within polymeric nanoparticles in the diabetes animal model. Eur J Pharm Sci.

[28] Wang H, Xu Z, Li Q, Wu J (2021). Application of metal-based biomaterials in wound repair. Eng Regen.

[29] Wilkinson H, Upson S, Banyard K, Knight R, Mace K, Hardman M (2019). Reduced iron in diabetic wounds: An oxidative stress-dependent role for STEAP3 in extracellular matrix deposition and remodeling. J Invest Dermatol.

[30] Holinstat M (2017). Normal platelet function. Cancer Metastasis Rev.

[31] He Z, Tang H, You X, Huang K, Dhinakar A, Kang Y, Yu Q, Wu J (2018). BAPTA-AM nanoparticle for the curing of acute kidney injury induced by ischemia/reperfusion. ingentaconnect. J Biomed Nanotechnol.

[32] Coger V, Million N, Rehbock C, Sures B, Nachev M, Barcikowski S, Wistuba N, Strauß S, Vogt PM (2019). Tissue concentrations of zinc, iron, copper, and magnesium during the phases of full thickness wound healing in a rodent model. Biol Trace Elem Res.

[33] Suslick KS, Choe SB, Cichowlas AA, Grinstaff MW (1991). Sonochemical synthesis of amorphous iron. Nature.

[34] Li M, Yang DP, Wang X, Lu J, Cui D (2013). Mixed protein-templated luminescent metal clusters (Au and Pt) for H2O2 sensing. Nanoscale Res Lett.

[35] Shibu ES, Ono K, Sugino S, Nishioka A, Yasuda A, Shigeri Y, Wakida SI, Sawada M, Biju V (2013). Photouncaging nanoparticles for MRI and fluorescence imaging in vitro and in vivo. ACS Nano.

[36] Ugga L, Romeo V, Tedeschi E, Brunetti A, Quarantelli M (2018). Superparamagnetic iron oxide nanocolloids in MRI studies of neuroinflammation. J Neurosci Methods.

[37] Yadav VK, Ali D, Khan SH, Gnanamoorthy G, Choudhary N, Yadav KK, Thai VN, Hussain SA, Manhrdas S (2020). Synthesis and characterization of amorphous iron oxide nanoparticles by the sonochemical method and their application for the remediation of heavy metals from wastewater. Nanomaterials.

[38] Xie J, Zheng Y, Ying JY (2009). Protein-directed synthesis of highly fluorescent gold nanoclusters. J Am Chem Soc.

[39] Rosales L, González JW (2013). Transport properties of two finite armchair graphene nanoribbons. Nanoscale Res Lett.

[40] Liu CL, Wu HT, Hsiao YH, Lai CW, Shih CW, Peng YK, Tang KC, Chang HW, Chien YC, Hsiao JK, Cheng JT, Chou PT (2011). Insulin-directed synthesis of fluorescent gold nanoclusters: Preservation of insulin bioactivity and versatility in cell imaging. Angew Chem Int Ed Engl.

[41] Yamashita T, Hayes P (2008). Analysis of XPS spectra of Fe2+ and Fe3+ ions in oxide materials. Appl Surf Sci.

[42] Razavi S, Seyedebrahimi R, Jahromi M (2019). Biodelivery of nerve growth factor and gold nanoparticles encapsulated in chitosan nanoparticles for schwann-like cells differentiation of human adipose-derived stem cells. Biochem Biophys Res Commun.

[43] Oviedo MJ, Quester K, Hirata GA, Vazquez-Duhalt R (2019). Determination of conjugated protein on nanoparticles by an adaptation of the Coomassie blue dye method. MethodsX.

[44] Lin YF, Cheng CW, Shih CS, Hwang JK, Yu CS, Lu CH (2016). MIB: metal ion-binding site prediction and docking server. J Chem Inf Model.

[45] Bazeera Ferdhous P, Aanandhalakshmi R, Ramya P, Vanavil B (2022). Scrutiny of metal ion binding sites in different alginate lyases through in silico analysis. Appl Biochem Biotechnol.

[46] Attri K, Chudasama B, Mahajan RL, Choudhury D (2023). Therapeutic potential of lactoferrin-coated iron oxide nanospheres for targeted hyperthermia in gastric cancer. Sci Rep.

[47] Shi Y (2023). Protective effects of smilax glabra roxb. Against lead-induced renal oxidative stress, inflammation and apoptosis in weaning rats and HEK-293 cells. Front Pharmacol.

[48] Singh I, Luxami V, Choudhury D, Paul K (2021). Synthesis and photobiological applications of naphthalimide–benzothiazole conjugates: cytotoxicity and topoisomerase IIα inhibition. RSC Adv.

[49] Odatsu T, Kuroshima S, Shinohara A, Valanezhad A, Sawase T (2021). Lactoferrin with Zn-ion protects and recovers fibroblast from H2O2-induced oxidative damage. Int J Biol Macromol.

[50] Yang X, Peng Q, Liu Q, Hu J, Tang Z, Cui L, Lin Z, Xu B, Lu K, Yang F (2017). Antioxidant activity against H2O2-induced cytotoxicity of the ethanol extract and compounds from Pyrola decorate leaves. Pharm Biol.

[51] Mirzaei M, Mirdamadi S, Safavi M (2019). Antioxidant activity and protective effects of Saccharomyces cerevisiae peptide fractions against H 2 O 2-induced oxidative stress in Caco-2 cells. J Food Meas Charact.

[52] Datta S, Choudhury D, Das A, Mukherjee DD, Dasgupta M, Bandopadhyay S, Chakrabarti G (2019). Autophagy inhibition with chloroquine reverts paclitaxel resistance and attenuates metastatic potential in human nonsmall lung adenocarcinoma A549 cells via ROS mediated modulation of β-catenin pathway. Apoptosis.

[53] Haldar S, Dru C, Choudhury D, Mishra R, Fernandez A, Biond S, Li Z, Shimada K, Arditi M, Bhowmick NA (2015). Inflammation and pyroptosis mediate muscle expansion in an interleukin-1β (IL-1β)-dependent manner. J Biol Chem.

[54] Ganguli A, Choudhury D, Chakrabarti G (2014). toxicology 2,4-Dichlorophenoxyacetic acid induced toxicity in lung cells by disruption of the tubulin-microtubule network. Toxicol Res.

[55] Ali OM, Bekhit AA, Khattab SN, Helmy MW, Abdel-Ghany YS, Teleb M, Elzoghby AO (2020). Synthesis of lactoferrin mesoporous silica nanoparticles for pemetrexed/ellagic acid synergistic breast cancer therapy. Colloids Surf B Biointerf.

[56] Choudhury D, Ganguli A, Dastidar DG, Acharya BR, Das A, Chakrabarti G (2013). Apigenin shows synergistic anticancer activity with curcumin by binding at different sites of tubulin. Biochimie.

[57] Togashi T, Naka T, Asahina S, Sato K, Takami S, Adschiri S (2011). Surfactant-assisted one-pot synthesis of superparamagnetic magnetite nanoparticle clusters with tunable cluster size and magnetic field sensitivity. Dalt Trans.

[58] Joseph C, Daniels A, Singh S, Singh M (2022). Histidine-tagged folate-targeted gold nanoparticles for enhanced transgene expression in breast cancer cells in vitro. Pharm.

[59] Maloň P, Bedna´rova´ L, Bedna´rova B, Bedna´rova´ B, Straka M, Krejč I ´ L, Luka´ L, Kumprecht L, Toma´ T, Kraus T, Ta M, Kuba´ K, Ova´ K, Ova´ O, Vladimi´ V, Baumruk V. Disulfide chromophore and its optical activity. Chirality. 2010;22:E47-E55. 10.1002/chir.20851 10.1002/chir.20851 21038396

[60] Stålhandske CMV, Stålhandske CI, Persson I, Sandström M, Jalilehvand F (2001). Crystal and solution structures of N, N-dimethylthioformamide-solvated copper(I), silver(I), and gold(I) ions studied by X-ray diffraction, X-ray absorption, and vibrational spectroscopy. Inorg Chem.

[61] Bock P, Nousiainen P, Elder T, Blaukopf M, Amer H, Zirbs R, Potthast A, Gierlinger N (2020). Infrared and Raman spectra of lignin substructures: Dibenzodioxocin. J Raman Spectrosc.

[62] Luber S (2013). Solvent effects in calculated vibrational Raman optical activity spectra of α-helices. J Phys Chem A.

[63] Sharma SK, Chio CH, Muenow DW. Raman spectroscopic investigation of ferrous sulfate hydrates. in 37th Annual Lunar and Planetary Science Conference 1078 (2006).

[64] Roeters SJ, Van Dijk CN, Torres-Knoop A, Backus EHG, Campen RK, Bonn M, Woutersen S (2013). Determining in situ protein conformation and orientation from the amide-I sum-frequency generation spectrum: theory and experiment. J Phys Chem A.

