# Supplementary material for: Integrated insulin-iron nanoparticles: a multi-modal approach for receptor-specific bioimaging, reactive oxygen species scavenging, and wound healing
Source: Discov Nano. 2024 May 30;19(1):96. doi: 10.1186/s11671-024-04024-6 (PMC11139842; doi:10.1186/s11671-024-04024-6)
Supplement: Supplementary file 1 — Additional file 1: Figure S1. Figure shows (a) FE-SEM image to study the surface morphology of synthesized (b) DLS for confirming the stability of the synthesized nano-formulation after 3 months (92 days). Figure S2. FTIR spectra to confirm the interaction between protein Insulin and iron after formation of nanoparticles (500–1600 cm-1). Figure S3. Median plots of (a) FeSO4 salt (b) Insulin for finding the y-intercept and m values to calculate Dm to determine the combination index of iron and insulin. Table S1. The table shows the % variation in mitochondrial reductase activity in MTT assay for determining cellular metabolism rate using HEKa cells. The cells were treated with varying concentrations; 1.5 µM, 7.5 µM, 15 µM, and 30 µM of Insulin, FeSO4, Insulin + FeSO4, and IFe(II)NPs for a duration of 24 h. The data was plotted as mean value ± SD of three independent experiments. Table S2. It shows the p values calculated for % change in cell viability after treatment with varying concentrations of Insulin, FeSO4, Insulin + FeSO4 and IFe(II)NPs. The data is considered to be statistically significant when p < 0.05. Table S3. It shows the p values calculated for % variation in wound diameter after treatment with 15 µM of Insulin, FeSO4, Insulin + FeSO4, and IFe(II)NPs. The data is considered to be statistically significant when p < 0.05. [file 11671_2024_4024_MOESM1_ESM.docx]

**Integrated Insulin-Iron Nanoparticles: A Multi-Modal Approach for Receptor-Specific Bioimaging, Reactive Oxygen Species Scavenging, and Wound Healing**

Komal Attri^1,4^, Bhupendra Chudasama^2,4*^, Roop L. Mahajan^3,4*^, Diptiman Choudhury^1,4^*

^1^Department of Chemistry and Biochemistry, Thapar Institute of Engineering and Technology, Patiala-147004, Punjab, India.

^2^Department of Physics and Material Sciences, Thapar Institute of Engineering and Technology, Patiala-147004, Punjab, India

^3^Department of Mechanical Engineering, Virginia Tech, Blacksburg, VA 24061, United States; Institute for Critical Technology and Applied Science, Virginia Tech, Blacksburg, VA 24061, United States.

^4^TIET-VT Centre of Excellence for Emerging Materials, Thapar Institute of Engineering and Technology, Patiala-147004 Punjab, India.

*****Corresponding E-mail: [diptiman@thapar.edu](mailto:diptiman@thapar.edu), [mahajanr@vt.edu](mailto:mahajanr@vt.edu), bnchudasama@thapar.edu

*Corresponding Phone: +91-8196949843 / +1-5402312597 / +91-9781966136


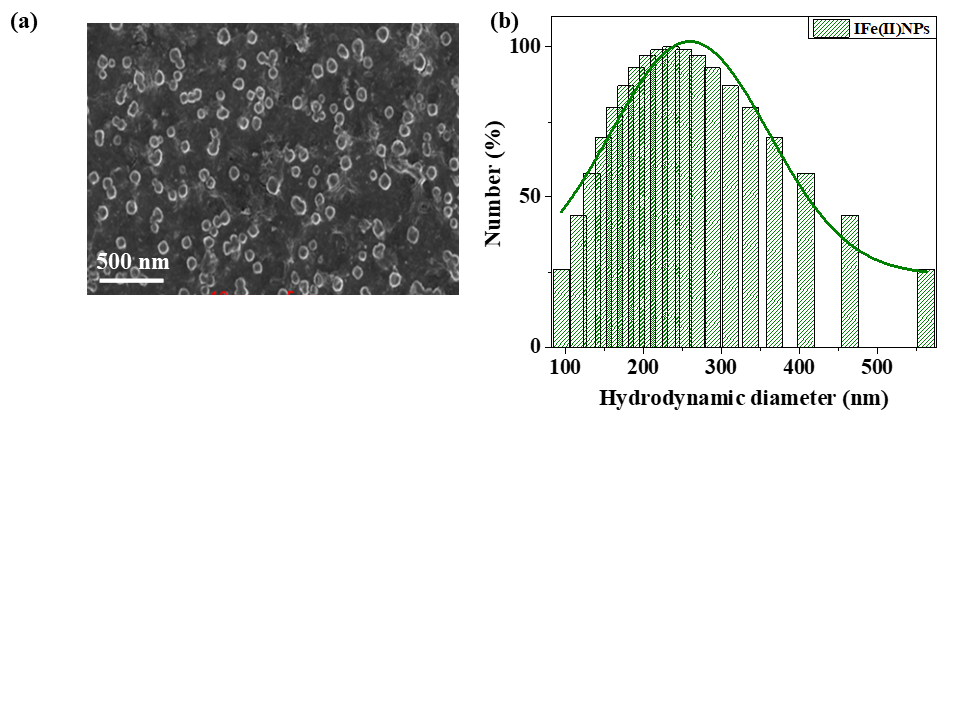


**Figure S1:** Figure shows (a) FE-SEM image to study the surface morphology of synthesized (b) DLS for confirming the stability of the synthesized nano-formulation after 3 months (92 days)


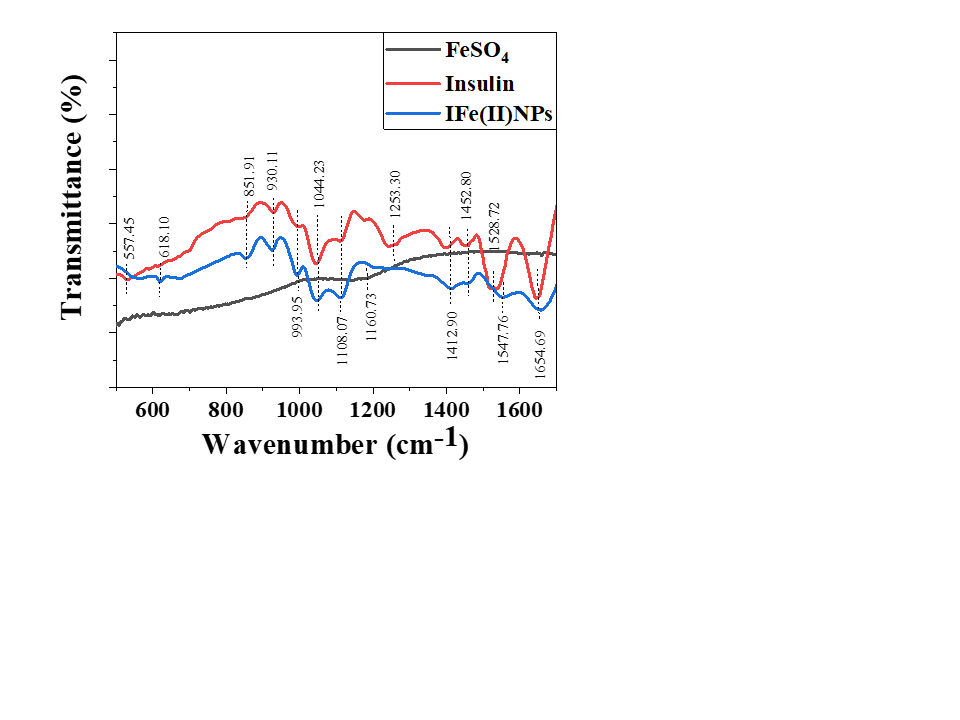


**Figure S2:** FTIR spectra to confirm the interaction between protein Insulin and iron after formation of nanoparticles (500 – 1600 cm^-1^)

**Figure S3:** Median plots of **(a)** FeSO_4_ salt **(b)** Insulin for finding the y-intercept and m values to calculate Dm to determine the combination index of iron and insulin.

**Table S1:** The table shows the % variation in mitochondrial reductase activity in MTT assay for determining cellular metabolism rate using HEKa cells. The cells were treated with varying concentrations; 1.5 µM, 7.5 µM, 15 µM, and 30 µM of Insulin, FeSO_4_, Insulin + FeSO_4,_ and IFe(II)NPs for a duration of 24 hours. The data was plotted as mean value ± SD of three independent experiments.

| **% change in mitochondrial reductase activity** | | | | |
| --- | --- | --- | --- | --- |
| **Dose** | **Insulin** | **FeSO_4_** | **FeSO_4_ + Insulin** | **IFe(II)NPs** |
| **1.5 µM** | 105.19 ± 6.25 % | 103.12 ± 8.00% | 104.42 ± 7.49 % | 114.60 ± 17.55 % |
| **7.5 µM** | 109.67 ± 5.05 % | 104.36 ± 2.31 % | 112.12 ± 2.33 % | 129.79 ± 4.16 % |
| **15 µM** | 113.86 ± 4.02 % | 108.14 ± 2.96 % | 116.72 ± 2.61 % | 136.28 ± 8.20 % |
| **30 µM** | 129.88 ± 2.89 % | 109.91 ±2.88 % | 123.77 ± 7.93 % | 145.13 ± 2.96 % |

**Table S2** It shows the p values calculated for % change in cell viability after treatment with varying concentrations of Insulin, FeSO_4_, Insulin + FeSO_4_ and IFe(II)NPs. The data is considered to be statistically significant when p < 0.05.

| **P value for checking the statistical significance of data** | | | | |
| --- | --- | --- | --- | --- |
| **Dose** | **Insulin** | **FeSO_4_** | **Insulin +FeSO_4_** | **IFe(II)NPs** |
| **1.5 µM** | 0.045211 | 0.519282 | 0.047125 | 0.049888 |
| **7.5 µM** | 0.043211 | 0.306961 | 0.009702 | 0.005798 |
| **15 µM** | 0.020066 | 0.041644 | 0.009702 | 0.000611 |
| **30 µM** | 0.001452 | 0.049845 | 0.004522 | 0.002223 |

**Table S3** It shows the p values calculated for % variation in wound diameter after treatment with 15 µM of Insulin, FeSO_4_, Insulin + FeSO_4,_ and IFe(II)NPs. The data is considered to be statistically significant when p < 0.05.

| **P value for checking the statistical significance of data** | | | | |
| --- | --- | --- | --- | --- |
| **Time** | **Insulin** | **FeSO_4_** | **Insulin + FeSO_4_** | **IFe(II)NPs** |
| **8h** | 0.000766 | 0.00844 | 0.000944 | 5.1E-05 |
| **16h** | 0.001854 | 0.020972 | 8.07E-05 | 1.18E-05 |
| **32h** | 0.000385 | 0.007966 | 0.001317 | 6.66E-05 |
